# Kidney shape statistical analysis: associations with disease and anthropometric factors

**DOI:** 10.1186/s12882-023-03407-8

**Published:** 2023-12-06

**Authors:** Marjola Thanaj, Nicolas Basty, Madeleine Cule, Elena P. Sorokin, Brandon Whitcher, Ramprakash Srinivasan, Rachel Lennon, Jimmy D. Bell, E. Louise Thomas

**Affiliations:** 1 Research Centre for Optimal Health, School of Life Sciences, University of Westminster, London, UK; 2 Calico Life Sciences LLC, South San Francisco, CA USA; 3 Wellcome Centre for Cell-Matrix Research, Division of Cell-Matrix Biology and Regenerative Medicine, School of Biological Sciences, Faculty of Biology Medicine and Health, Manchester Academic Health Science Centre, The University of Manchester, Manchester, UK; 4 Department of Paediatric Nephrology, Royal Manchester Children’s Hospital, Manchester University Hospitals NHS Foundation Trust, Manchester Academic Health Science Centre, Manchester, UK

**Keywords:** Magnetic resonance imaging, Kidney volume, 3D mesh-derived phenotype, Statistical parametric maps, Statistical shape analysis, Chronic kidney disease, Type-2 diabetes, Hypertension

## Abstract

**Background:**

Organ measurements derived from magnetic resonance imaging (MRI) have the potential to enhance our understanding of the precise phenotypic variations underlying many clinical conditions.

**Methods:**

We applied morphometric methods to study the kidneys by constructing surface meshes from kidney segmentations from abdominal MRI data in 38,868 participants in the UK Biobank. Using mesh-based analysis techniques based on statistical parametric maps (SPMs), we were able to detect variations in specific regions of the kidney and associate those with anthropometric traits as well as disease states including chronic kidney disease (CKD), type-2 diabetes (T2D), and hypertension. Statistical shape analysis (SSA) based on principal component analysis was also used within the disease population and the principal component scores were used to assess the risk of disease events.

**Results:**

We show that CKD, T2D and hypertension were associated with kidney shape. Age was associated with kidney shape consistently across disease groups. Body mass index (BMI) and waist-to-hip ratio (WHR) were also associated with kidney shape for the participants with T2D. Using SSA, we were able to capture kidney shape variations, relative to size, angle, straightness, width, length, and thickness of the kidneys, within disease populations. We identified significant associations between both left and right kidney length and width and incidence of CKD (hazard ratio (HR): 0.74, 95% CI: 0.61–0.90, *p* < 0.05, in the left kidney; HR: 0.76, 95% CI: 0.63–0.92, *p* < 0.05, in the right kidney) and hypertension (HR: 1.16, 95% CI: 1.03–1.29, *p* < 0.05, in the left kidney; HR: 0.87, 95% CI: 0.79–0.96, *p* < 0.05, in the right kidney).

**Conclusions:**

The results suggest that shape-based analysis of the kidneys can augment studies aiming at the better categorisation of pathologies associated with chronic kidney conditions.

**Supplementary Information:**

The online version contains supplementary material available at 10.1186/s12882-023-03407-8.

## Introduction

The incidence of conditions such as chronic kidney disease (CKD), type-2 diabetes (T2D), and hypertension are rising and are amongst the leading causes of death globally [1]. The prevalence of CKD, which increases with age, is significantly common among older people, with an adverse effect on longevity [2]. Progression of CKD is generally tracked as a gradual decline in glomerular filtration rate (GFR), however there are suggestions that changes in kidney volume occur much earlier in the disease process and can accurately predict disease [3, 4]. Changes in kidney volume, as well as kidney length and structure, have additionally been reported in metabolic diseases including obesity, T2D, and hypertension, these act as drivers of CKD progression reducing kidney function [5–7].

Recently the automated segmentation and measurement of kidney volume from magnetic resonance imaging (MRI) has become more commonplace, enabling rapid measurement and the ability to obtain detailed anatomical information [8]. While this advance has enhanced our understanding of the kidney at a population level, additional knowledge regarding morphological changes and regional variation in response to particular conditions are still lacking.

Three-dimensional (3D) mesh-derived phenotypes capture additional information related to morphological and regional organ variation using statistical parametric maps (SPMs) and may be used to map more subtle differences between a healthy and diseased state. A similar approach is statistical shape analysis (SSA), which can be used to transform the spatially correlated data into a smaller number of principal components and characterise variations in organ shape across a population. These morphometric analyses offer a way to model the human body non-invasively and have been widely used to model bones [9, 10], abdominal organs [11–13], the brain [14, 15], the heart [16, 17] and the aorta [18, 19]. However, they have been less frequently applied to abdominal organs, despite known morphological changes occurring in clinical conditions [3].

The objectives of this study were to (1) identify factors associated with variation in kidney shape in a region-specific manner, through computational image analysis, and assess whether this analysis can be used to identify morphological variation associated with anthropometric and clinical conditions CKD, T2D, and hypertension; and (2) investigate whether the emerging 3D kidney mesh-derived phenotypes can add to the prediction of disease outcomes.

## Methods

### Data

Full details regarding the UK Biobank abdominal MRI acquisition protocol have previously been reported [20]. Briefly, the data included here focused on the neck-to-knee Dixon MRI acquisitions involving six overlapping series that were positioned automatically following an initial selection made by the radiographer. All data were processed and segmented using automated methods [8].

Participant data from the UK Biobank cohort was obtained through UK Biobank Access Application number 44584. The UK Biobank has approval from the North West Multi-Centre Research Ethics Committee (REC reference: 11/NW/0382). All methods were performed in accordance with the relevant guidelines and regulations, and informed consent was obtained from all participants. Researchers may apply to use the UKBB data resource by submitting a health-related research proposal that is in the public interest. Additional information may be found on the UK Biobank researchers and resource catalogue pages (www.ukbiobank.ac.uk).

### Quality control

Participants with missing clinical, anthropometric, or biological data, as well as those with Dixon MRI datasets that did not have full anatomical coverage, were excluded from the study (i.e. organs with zero volumes). More specifically, we removed 5,272 data that were missing anthropometric, biological and lifestyle variables that are necessary for analyses. To ensure comprehensive anatomical coverage, we also discarded kidney segmentations with volumes less than 30 ml from our analysis, resulting in the exclusion of further 305 data. For consistency in the sample size, participants with missing data or segmentations below the lower limit threshold for one kidney were excluded from the study even if the other kidney had full coverage. We further performed quality control by visually inspecting potential outliers in the 3D kidney mesh-derived phenotype to potentially identify extremely high values, falling outside from randomly selected quantiles (0.1% and 99.9%), however no further exclusion was required. Supplementary Fig. S1 shows a flow diagram of the quality control process and resulting study population. Overall, from the initial 44,445 participants, data from of 5,577 participants did not pass quality control and were excluded from the final analysis (12.6% of the dataset excluded, of which 47% were male, 64.7 ± 7.8 years old, with a body mass index (BMI) 26.3 ± 4.5 kg/m^2^ [mean ± standard deviation (SD)]), leaving a final dataset of 38,868 participants.

### Image registration and mesh construction

The process for organ template construction has been previously detailed in [21, 22]. Here, we constructed a template using the kidney segmentations from a sex-balanced European ancestry cohort of 200 participants. The characteristics of the template population are provided in Supplementary Table S2. We then constructed 3D surface meshes from the template image and all participants’ segmentations using the marching cubes algorithm. These meshes were subsequently smoothed using a Laplacian filter [23]. Supplementary Fig. S2 illustrates a brief diagram for the construction of average kidney template meshes.

The registration process we employed has been previously outlined in [22]. In brief, we initially used rigid registration to remove the position and orientation difference between all participant-specific surfaces and template surfaces. We then registered the template to the participant’s segmentations via affine and non-rigid registration. The template mesh was then propagated to each participant mesh using the deformation fields obtained from the non-rigid registration. Hence, all surface meshes are parameterised with the same number of vertices (approximately 4,000) ensuring each vertex was anatomically accurate and consistent across all participants while preserving the size and shape information for subsequent analysis. All the steps for the template-to-subject registration were performed using the Image Registration Toolkit (IRTK) (https://biomedia.doc.ic.ac.uk/software/irtk).

To determine the regional outward or inward adaptations in kidney surface relative to an average kidney shape for each participant, the surface-to-surface (S2S) distance, the 3D mesh derived phenotype was measured. This measurement involved computing the signed distance between each vertex in the template mesh and each corresponding vertex in the subjects' mesh. In this context, positive distances signify outward expansion in the subject’s vertices when compared to the template vertices, while negative distances indicate inward shrinkage in the subject’s vertices [22].

### Mass univariate regression analysis

Associations between S2S distances and anthropometric variables were estimated using a linear regression framework. The linear regression model is expressed as follows:

$$Y=X\beta +\epsilon ,$$

where, Y is a ${n}_{s}$ × ${n}_{v}$ matrix containing ${n}_{s}$ subjects from a sample of the population under study and ${n}_{v}$ is the number of voxels in the mesh, X is the ${n}_{s}$ × p design matrix of p known covariates (including the intercept) and the relevant variables for each subject. X is related to Y by the vector of the estimated regression coefficients $\widehat{\beta }$. Finally $\epsilon$ is a ${n}_{s}$ × ${n}_{v}$ matrix which is independent and identically distributed across the subjects and is assumed to be a zero-mean Gaussian process [24]. We applied threshold-free cluster enhancement (TFCE) [25] and permutation testing to assess the associations between S2S distances and anthropometric variables, and derive the *p*-values associated with each regression coefficient following adjustment for relevant covariates with the correction to control the false discovery rate (FDR), as previously described [21].

Specifically, we performed an SPM framework, mass univariate regression (MUR) analysis using a refined version of the R package *mutools3D *[26] adjusted for multiple comparisons by applying the FDR procedure using the Benjamini–Hochberg method [27] to all the TFCE-derived *p*-values for each vertex and each model using 1,000 permutations. The estimated regression coefficients $\widehat{\beta }$ for each of the relevant covariates and their related TFCE-derived *p*-values after correction for multiple testing, were then displayed at each vertex in the mesh on the whole 3D kidney anatomy, providing the spatially-distributed associations. The MUR model for deriving associations between clinical conditions and a 3D phenotype is outlined in supplementary Fig. S3.

To determine which factors were associated with kidney shape and size, we fitted a linear regression model for each vertex with age, sex, ethnicity, BMI, waist-to-hip ratio (WHR), alcohol drinker status, smoking status, ibuprofen medication, sodium in urine, urea and clinical conditions including CKD, T2D, and hypertension as predictors, and applied a correction to control the FDR. It is well known that the number of nephrons decreases with age which can contribute to the decline in kidney function [4]. Hence, to investigate whether there was a stronger relationship in terms of accelerating change between age and CKD, T2D, and hypertension we included interaction terms between age and all clinical conditions. We then included interaction terms between BMI and T2D, and between WHR and T2D, as markers of obesity, to investigate whether there is a change in kidney function caused by hyperfiltration. We further performed a sex-stratified analysis, producing two models for male and female separately, using all the aforementioned variables apart from sex. All continuous variables, including the S2S distances, were standardised with units in one SD, prior to being included in the regression models.

### Statistical shape analysis

Statistical shape analysis (SSA) is a technique defined by the variation of the size and shape (represented using a surface mesh) across participants. Principal component analysis (PCA) is a widely explored dimensionality reduction approach applied in SSA for constructing 3D statistical shape models [28, 29]. The principal directions of variation, also called modes of variation, could be represented by eigenvectors calculated from PCA. This technique finds a new coordinate system that describes the input data so that the greatest variance of the data lies on the first coordinate (the first principal component mode), the second-largest variance of the data that is orthogonal to the first component mode on the second coordinate, and so on. In this study, we initially performed PCA on the coordinate system and the S2S distances of the full cohort (*N* = 38,868), to explore whether these coefficients for the principal components known as shape parameters can characterise variations in kidney shape across the population.

### Survival analysis

Cox proportional hazards models were used to assess the risk of disease outcomes that occurred after the imaging visit. These models were adjusted for relevant covariates to account for potential confounding factors. To reduce the dimensionality of the 3D mesh-derived phenotype, we performed SSA by computing the PCA on the S2S distances across all participants within each disease cohort and extracted the principal component (PC) scores.

To determine the unique contribution of each kidney measurement and ensure robust statistical significance of associations, we employed separate models for each kidney (left and right). This approach allowed us to account for potential confounding and effectively capture the specific associations between the kidney measures and disease outcomes. In these separate models, we adjusted for a comprehensive set of covariates including age, sex, ethnicity, body mass index, waist-to-hip ratio, alcohol intake frequency, smoking status, ibuprofen medication, sodium in urine, urea, kidney volume and the first four principal component scores of the S2S distances.

Disease outcomes and dates of the first occurrence of CKD, T2D, and hypertension were defined based on a combination of hospital records, primary care records, self-report, and death records (see Disease Definitions in supporting information and supplementary Table S1). Time-to-event was censored at the first event for each outcome, death, or last recorded follow-up (10th of February 2022), with a median of 3.7 years follow-up period since the imaging visit. Participants with an event recorded prior to the imaging visit were excluded.

Model summaries are reported as hazard ratios with 95% confidence intervals (CI). To control for multiple testing, the FDR procedure was estimated from the *p*-values, and a threshold of FDR < 0.05 determined significance.

### Phenotype definitions

Anthropometric measurements including age, BMI, waist and hip circumferences, and systolic and diastolic blood pressure were taken at the UK Biobank imaging visit, and ethnicity was defined based on the self-reported ethnic background at the initial assessment visit (field: 21,000). For the purpose of our analysis we categorised ethnic background as follows: 0 for "White" and 1 for any other ethnic background (due to small numbers of non-white participants in this dataset (3.1%)). Sex was self-reported and included those recorded by the NHS and those obtained at the initial assessment visit (field: 31).

Biological samples for serum creatinine (field: 30,700) were measured in $millimole/L$, sodium in urine (field: 30,530) in $millimole/L$ and urea (field: 30,670) in $millimole/L$ units were taken on the initial assessment visit. Estimated glomerular filtration rate (eGFR) was calculated based on the CKD-EPI creatinine Eq. (2009) [30] as follows:

$$eGFR=141\times min(Scr/\kappa , 1{)}^{\alpha } \times max(Scr/\kappa, 1{)}^{-1.209}\times 0.99{3}^{Age} \left[\times\,1.018\, if\, female\right],\left[\times\, 1.159\, if\, black\right].$$

where $Scr$ is serum creatinine in converted into $mg/dL$ units, $\kappa$ is 0.7 for females and 0.9 for males, $\alpha$ is − 0.329 for females and − 0.411 for males, $min$ is the minimum of Scr/κ or 1, and $max$ is the maximum of Scr/κ or 1. In the aforementioned equation, ethnicity for “Black” was defined based on the continental genetic ancestry for “African” ancestry (https://pan.ukbb.broadinstitute.org) and if missing from the self-reported ethnic background (field: 21,000) for “Black or Black British”.

Questionnaire information from the UK Biobank imaging visit was used to determine alcohol intake frequency (field: 1558), smoking status (field: 20,116), and usage of ibuprofen medication (field: 6154, field: 20,003). For our analysis, we categorised alcohol intake frequency as 1 for “Daily or almost daily” and 0 otherwise and smoking status as 1 for”Current” and 0 for “Previous” and “Never”. It should be noted that the UK Biobank initial assessment visit preceded the imaging visit by 9 ± 1.7 years.

### Disease definitions

We selected diseases known to be associated with kidney health, and those previously associated with changes in kidney volume [8]. In part, this was also guided by the number of patients available in the UK Biobank with diseases of interest. We included participants with CKD, and T2D as well as participants with hypertension (see Disease Definitions in supporting information and supplementary Table S1).

## Results

### Study population characteristics

Of the cohort of 38,868 participants 96.9% were white and 48.3% male, with an age range between 44 and 82 years, and a mean BMI 26.5 ± 4.3 kg/m^2^ (supplementary Table S3). We identified 1,134 participants with CKD (581F/553 M), age 69 ± 6.9 years and a BMI 27.8 ± 4.7 kg/m^2^, 2,054 participants with T2D from which 66% were male, age 66.6 ± 7.3 years and BMI 29.7 ± 5.2 kg/m^2^ and 14,113 participants with hypertension of which 58% male; age 66.8 ± 7.2 years; BMI 28 ± 4.6 kg/m^2^.

From all 1,134 participants with CKD, defined by an eGFR below 60 ml/min/1.73 m2, codes for chronic kidney disease, kidney dialysis, and kidney transplantation operation codes (supplementary Table S1), 466 (41.1%) participants had an eGFR below 60 ml/min/1.73 m2 (53.8 ± 6.1, mean ± SD; 21.2—60 ml/min/1.73 m2, range) and 793 participants were identified based on the codes for diagnosis. Hence, from the participants diagnosed with CKD (*N* = 793), only 125 of them also had eGFR levels below 60 ml/min/1.73 m2 (supplementary Fig. S4). From the 14,113 participants with hypertension defined as self-reported of hypertensive medication, or a prior diagnosis of hypertension, or mean blood pressure ≥ 140/90 mmHg (see Disease Definitions in supporting information and supplementary Table S1), only 4,326 (30.7%) had mean blood pressure measurements ≥ 140/90 mmHg (supplementary Fig. S5).

### Associations with anthropometric traits and disease

**Fig. 1:**
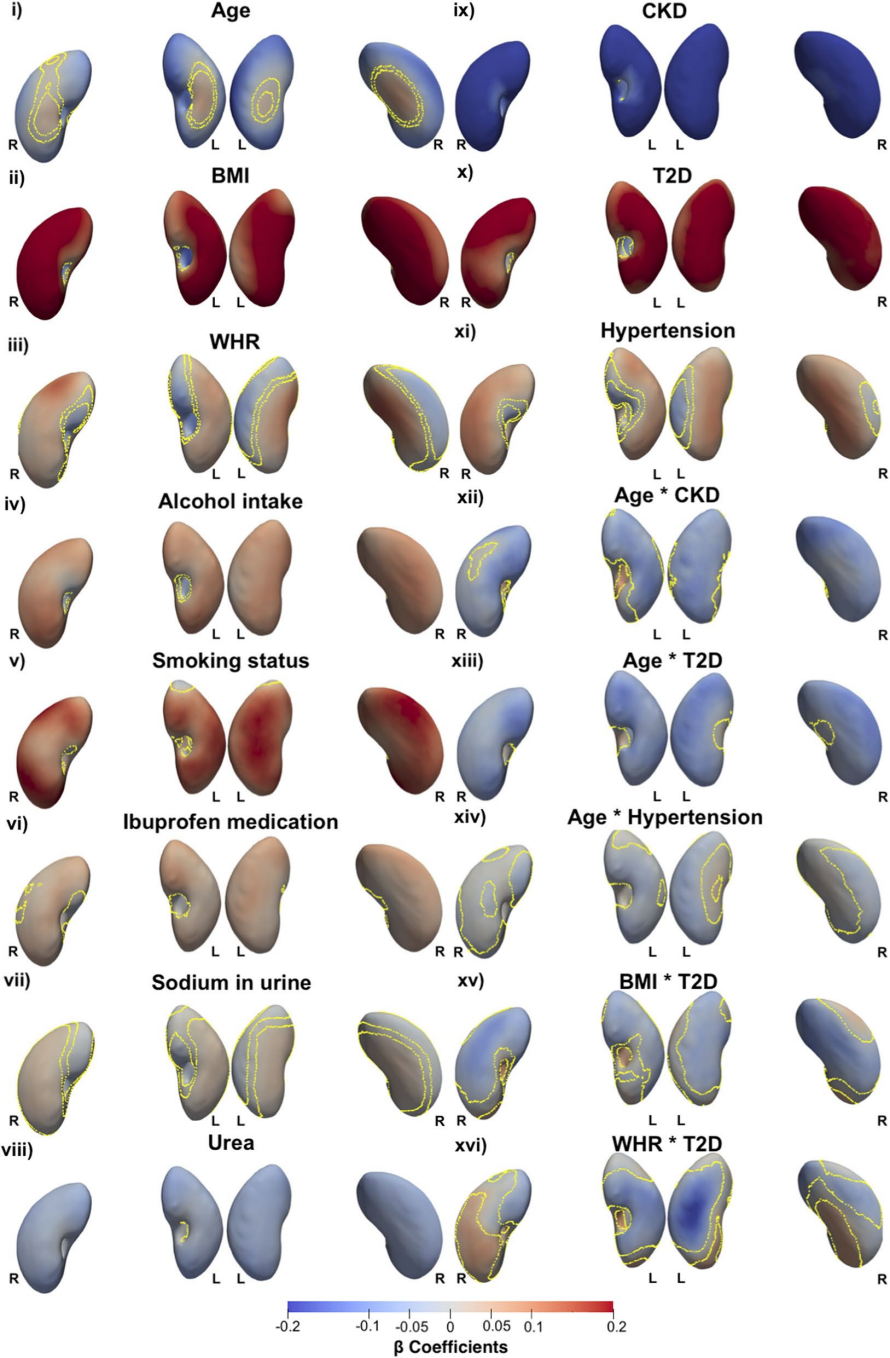
Three-dimensional statistical parametric maps (SPMs) of kidney morphology, projections are anterior and posterior views for both left (L) and right (R) kidneys in both anterior (left plots) and posterior (right plots) views. The SPMs show the local strength of association for each covariate in the model (**i** - **xvi**) with S2S distances on the full cohort (*N*   = 38,868). Yellow contour lines indicate the boundary between statistically significant regions (*p* < 0.05) after correction for multiple testing, with positive associations in bright red and negative associations in bright blue. The standardised regression coefficients ($\widehat{\beta }$) are shown with units in standard deviations for each covariate

We proceeded to register the 200 healthy participant template on the full cohort (*N* = 38,868), computing S2S distances between the template and surface of each individual kidney mesh, and performed MUR analysis to generate SPMs, adjusting for all relevant covariates. A summary of the model for the whole cohort, representing the standardised regression coefficients for each covariate and the significance areas on the kidney, is provided in Tables 1, 2, and supplementary Figs. S6, S7. The SPMs that represent associations between S2S distances and the anthropometric measurements are shown in Fig. 1.

**Table 1:** Significance areas for covariates in the MUR model for the anthropometric variables (*N* = 38,868) of the model for the left kidney. The total area has been split into areas of positive, negative, and total associations. The standardised regression coefficients ($\widehat{\beta }$) are presented as median (interquartile range—IQR) across all vertices of the left kidney surface and the significance areas as a percentage (%) of the vertices with statistically significant associations

Left Kidney	Standardised $\widehat{\beta }<0$		Standardised $\widehat{\beta }>0$		Total
	Beta coefficients	Significance area	Beta coefficients	Significance area	Significance area
**Age**	-0.10 (0.07)	89.84%	0.03 (0.02)	5.5%	95.32%
**BMI**	-0.10 (0.07)	14.79%	0.20 (0.09)	85%	99.79%
**WHR**	-0.04 (0.04)	37.44%	0.04 (0.03)	54.98%	92.42%
**Alcohol intake frequency**	-0.06 (0.03)	14.47%	0.07 (0.02)	84.43%	98.9%
**Smoking status**	-0.05 (0.01)	3.88%	0.12 (0.05)	84.36%	88.24%
**Ibuprofen medication**	-0.04 (0.03)	0.5%	0.04 (0.02)	84.27%	84.77%
**Sodium in urine**	-0.02 (0.02)	31.05%	0.02 (0.01)	48.81%	79.86%
**Urea**	-0.06 (0.01)	88.84%	0.02 (0.02)	6.6%	95.43%
**CKD**	-0.33 (0.11)	91.6%	0.09 (0.04)	3.74%	95.34%
**T2D**	-0.06 (0.02)	1.78%	0.20 (0.08)	85.5%	87.28%
**Hypertension**	-0.03 (0.02)	19.59%	0.06 (0.04)	67.44%	87.03%
Age * CKD	-0.06 (0.03)	67.31%	0.06 (0.02)	9.54%	76.85%
Age * T2D	-0.06 (0.03)	89.98%	0.002 (0.002)	0.07%	90.05%
Age * Hypertension	-0.03 (0.02)	71.39%	0.02 (0.003)	1%	72.4%
BMI * T2D	-0.05 (0.02)	59%	0.05 (0.02)	5.09%	64.09%
WHR * T2D	-0.07 (0.04)	49.93%	0.05 (0.02)	8.97%	58.9%

**Table 2:** Significance areas for covariates in the MUR model for the anthropometric variables (*N* = 38,868) of the model for the right kidney. The total area has been split into areas of positive, negative, and total associations. The standardised regression coefficients ($\widehat{\beta }$) are presented as median (interquartile range—IQR) across all vertices of the left kidney surface and the significance areas as a percentage (%) of the vertices with statistically significant associations

Right Kidney	Standardised $\widehat{\beta }<0$		Standardised $\widehat{\beta }>0$		Total
	Beta coefficients	Significance area	Beta coefficients	Significance area	Significance area
**Age**	-0.07 (0.05)	78.32%	0.03 (0.03)	12.43%	90.75%
**BMI**	-0.14 (0.03)	12.46%	0.23 (0.07)	87.35%	99.81%
**WHR**	-0.05 (0.07)	27.26%	0.04 (0.05)	62.82%	90.08%
**Alcohol intake frequency**	-0.05 (0.02)	12.77%	0.07 (0.02)	85.77%	98.54%
**Smoking status**	-0.06 (0.02)	2.13%	0.13 (0.05)	92.17%	94.3%
**Ibuprofen medication**	-0.01 (0.01)	0.48%	0.04 (0.01)	83.21%	83.68%
**Sodium in urine**	-0.02 (0.02)	35.36%	0.02 (0.01)	48.78%	71.61%
**Urea**	-0.06 (0.02)	91.78%	0.02 (0.02)	4.91%	96.69%
**CKD**	-0.30 (0.13)	94.66%	0.09 (0.03)	2.54%	97.29%
**T2D**	-0.06 (0.02)	6.97%	0.20 (0.05)	87.76%	94.73%
**Hypertension**	-0.04 (0.03)	10.76%	0.07 (0.05)	79.76%	90.51%
Age * CKD	-0.06 (0.04)	72.54%	0.06 (0.02)	3.74%	76.28%
Age * T2D	-0.06 (0.03)	89.84%	-	-	89.84%
Age * Hypertension	-0.02 (0.01)	40.97%	0.01 (0.01)	0.19%	41.16%
BMI * T2D	-0.07 (0.04)	59.63%	0.06 (0.03)	14.23%	73.86%
WHR * T2D	-0.05 (0.02)	22.28%	0.05 (0.03)	28.56%	50.84%

S2S distances were negatively associated with age, showing an inward shape variation observed in the inferior and superior areas of both kidneys. There was also a positive association between age and S2S distances, shown in the anterior and posterior areas of both kidneys. BMI and WHR had statistically significant positive associations with S2S distances, covering 85% and 55% of the left kidney surface and 87.3% and 62.8% of the right kidney surface, respectively. Alcohol intake frequency, smoking status and use of ibuprofen medication showed a statistically significant outward shape variation in both kidneys. We further examined the associations between S2S distances and biological markers including sodium in urine and urea. Sodium in urine showed a statistically significant positive association with S2S distances in both kidneys, while urea showed an inward shape variation with S2S distances in the both kidneys.

A diagnosis of CKD was associated with S2S distances with an inward shape variation in both kidneys. T2D was positively associated with S2S distances in both kidneys and hypertension showed an outward shape variation in S2S distances covering 67.4% of the left kidney and 79.8% of the right kidney. To facilitate a clearer interpretation, we have provided a table with the unstandardised (raw) regression coefficients to estimate the shape variations associated with the disease state in mm, based on the standardised coefficients and the median SD of the S2S distances across all vertices (supplementary Table S4). By considering that the median SD value for the S2S distances of the left kidney is 3.02 mm and for the right kidney is 2.95 mm, we showed that a diagnosis of CKD was associated with a median inward shape variation of -1 mm for the left kidney and -0.89 mm for the right kidney. T2D exhibited a positive association with S2S distances, with a median of 0.6 mm for the left kidney and 0.59 mm for the right kidney. Hypertension demonstrated a positive association with S2S distances, resulting in a median outward shape variation of 0.18 mm in the left kidney and 0.15 mm in the right kidney.

**Fig. 2:**
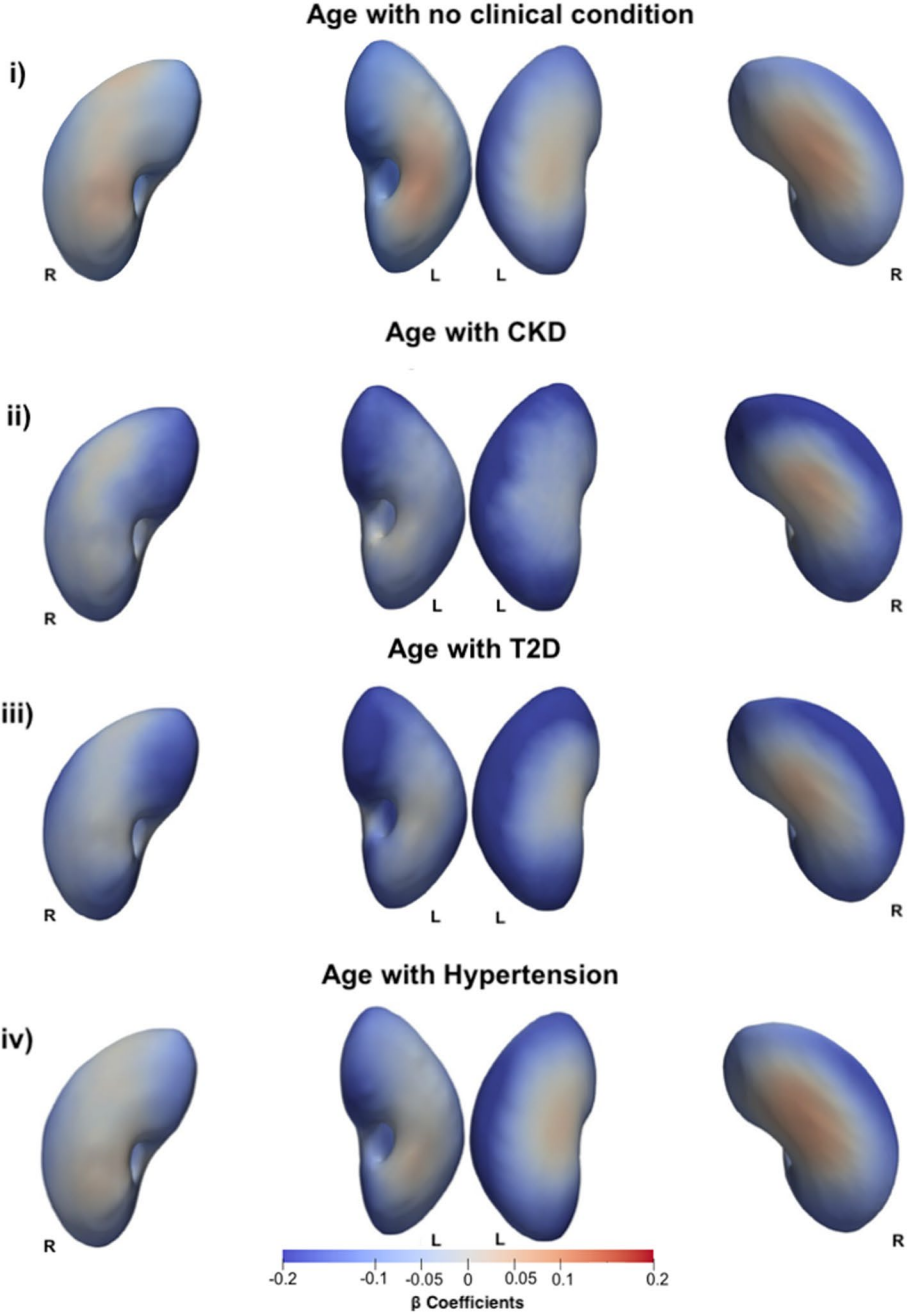
Three-dimensional statistical parametric maps (SPMs) of kidney morphology, projections are anterior (left plots) and posterior (right plots) views for both left (L) and right (R) kidneys. The SPMs show the local rate of change as a function of age for S2S distances in participants **i**) without CKD, T2D, or hypertension, **ii**) with CKD, referred to the sums of the regression coefficients for Age and Age*CKD, **iii**) with T2D, referred to the sums of the regression coefficients for Age and Age*T2D, and **iv**) with Hypertension, referred to the sums of the regression coefficients for Age and Age*Hypertension, in the full cohort (*N*  =  38,868). Positive associations are in red and negative associations are in blue. Regression coefficients ($\widehat{\beta }$) are shown with units in standard deviations for each covariate

**Fig. 3:**
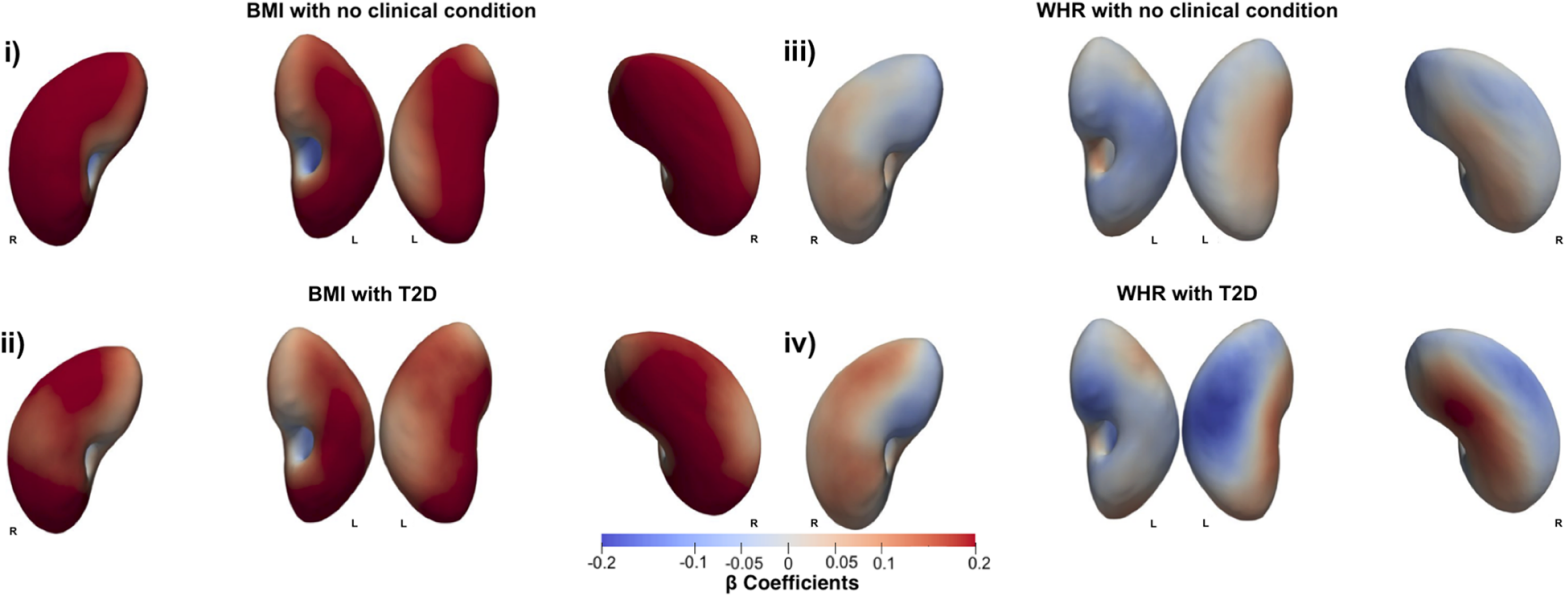
Three-dimensional statistical parametric maps (SPMs) of kidney morphology, projections are anterior (left plots) and posterior (right plots) views for both left (L) and right (R) kidneys. **i**) The SPMs show the local rate of change as a function of BMI for S2S distances in participants without T2D and **ii**) with T2D, referred to the sums of the regression coefficients for BMI and BMI*T2D, **iii**) SPMs show the local rate of change as a function of WHR for S2S distances in participants without T2D and **iv**) with T2D, referred to the sums of the regression coefficients for WHR and WHR*T2D, on the full cohort (*N* = 38,868). Positive associations are in red and negative associations in blue. Regression coefficients ($\widehat{\beta }$) are shown with units in standard deviations for each covariate

We undertook further analysis to determine whether there was an interaction between all clinical conditions and age, adjusted for all covariates in the model. We observed a stronger relationship between age and S2S distances with a median inward shape variation of -0.16 in CKD participants, compared with -0.10 in non-CKD participants in the left kidneys and median shape variation in S2S distances of -0.13 in CKD participants compared with -0.07 in non-CKD participants in the right kidney for the main effect of age (Tables 1, 2, and Fig. 1). The association between age and S2S distances in participants with CKD and without a clinical condition are directly compared in Fig. 2i and ii. Participants with T2D (Fig. 2iii) display a stronger relationship with S2S distances with a median inward shape variation of -0.16 in the left kidney and a median inward shape variation of -0.13 in the right kidney with increasing age. Participants with hypertension (Fig. 3iv) showed a median inward shape variation in S2S distances with increasing age of -0.13 in the left kidney and -0.09 in the right kidney.

We further investigated whether there was an interaction between T2D and BMI as well as WHR adjusted for all covariates in the model (Tables 1, 2, and Fig. 1). BMI in participants with T2D showed a stronger relationship with S2S distances with a median outward shape variation 0.05 covering only a small significance area of only 5.1% of the left kidney and a median outward shape variation of 0.06 covering an area of 14.2% of the right kidney in addition to the median outward shape variation of 0.20 in the left kidney and 0.23 in the right kidney for the main effect of BMI. However, we observed a stronger relationship in S2S distances with a median inward shape variation covering a significance area of 59% in the left kidney and 59.6% in the right kidney, for the main effect of BMI (Fig. 3i, ii).

From the interaction between WHR and T2D (Tables 1, 2, and Fig. 1), we observed a stronger relationship with S2S distances with a median outward shape variation of 0.05 in the both kidneys, in addition to the median shape variation of 0.04 in the both kidneys for the main effect of WHR. However, we also found a stronger relationship in S2S distances with a median inward shape variation of -0.07 in addition to the median shape variation of -0.04 in the left kidney and -0.05 in addition to the median shape variation of -0.05 in the right kidney for the main effect of WHR (Fig. 3iii, iv).

### Associations by sex

We performed MUR analysis to explore the associations between kidney shape and anthropometric and disease traits separated by sex, adjusting for all relevant covariates excluding sex. A summary of the model for the whole cohort, representing the standardised regression coefficients and the significance areas on the kidney is provided in supplementary Tables S5 and S6, and the histograms showing the statistically significant standardised regression coefficients is provided in supplementary Figs. S8 and S9. The SPMs that represent associations between S2S distances and the anthropometric measurements are shown in supplementary Fig. S10 for male (*N* = 18,855) and Fig. S10 for female participants (*N* = 20,013).

We observed similar associations with the model including the full cohort for both sexes. However, we noticed that the WHR in both the left and right kidneys showed more statistically significant negative associations with S2S distances in male participants and more statistically significant positive associations in female participants (supplementary Tables S5 and S6).

We also observed the interaction between age and all disease outcomes, BMI and T2D as well as WHR and T2D for male and female participants, separately. Female participants with CKD showed a strong relationship with S2S distances with a median inward shape variation of -0.09 in the left kidney and -0.08 in the right kidney, in addition to the median shape variation of -0.10 in the left kidney and -0.08 in the right kidney for the main effect of age. While, male participants with CKD showed a strong relationship with S2S distances with a median inward shape variation of -0.10 in the left kidney and -0.11 in the right kidney, in addition to the median shape variation of -0.10 in the left kidney and for -0.08 in the left kidney the main effect of age. We further found that both male and female participants with T2D showed a strong relationship with S2S distances showing a greater significance area in male participants with inward shape variations observed in the both kidneys, for the main effect of age. Furthermore, male participants with hypertension showed a stronger relationship with S2S distances for the main effect of age, with inward shape variations in both kidneys.

Interactions between BMI and T2D showed a strong relationship with S2S distances in both sexes although a greater significance area was observed in men with a median inward shape variation for both kidneys. Finally, male participants with T2D showed a strong relationship with S2S distances for the main effect of WHR, with outward shape variations observed in both kidneys covering a small significance area, while female participants with T2D showed an inward shape variation in the left kidney for the main effect of WHR.

### Statistical shape analysis

**Fig. 4:**
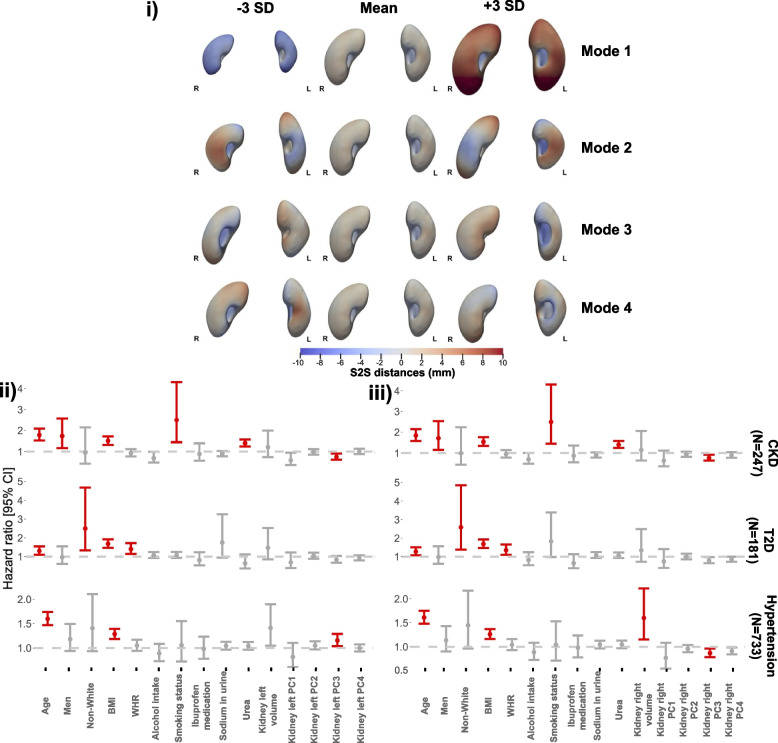
**i**) The first four modes of shape variation for the kidneys of the full cohort (*N* = 38,868). The mean shape and the shape at the ± three SD are displayed for each mode showing the S2S distance variation in mm. The kidney shape variations are shown in the anterior views for both left (L) and right (R) kidneys. **ii**) Hazard ratios and 95% CIs for the three outcomes: CKD, T2D, and hypertension for the left kidney. **iii**) Hazard ratios for the three disease outcomes for the right kidney adjusted for age, sex, ethnicity, body mass index, waist-to-hip ratio, sodium in urine, urea, alcohol intake frequency, smoking status, ibuprofen medication uptake, kidney volume and the first 4 principal component scores for the S2S distances. Significant associations for *p* < 0.05 are shown in red and non-significant associations in grey

To visualise shape variation across a population, we computed the PCA of the coordinate system including the S2S distances from the full cohort (*N* = 38,868). The first four modes of kidney size and shape variation from the PCA are presented as -3 SD, mean and + 3 SD (Fig. 4i and supplementary video S1).

The principal components are mathematically derived, so they do not necessarily correspond to physical features but they can be broadly interpreted by visualising extreme values along each axis (± 3 SD). The percentage of shape variation explained by the first ten modes of PCA for both left and right kidneys on the full cohort is presented in supplementary Fig. S12. The first principal component accounting for 39.5% of the shape variation for the left kidney and 41.2% for the right kidney, appeared to correspond best to the size of the kidney. The second principal component appeared to correspond to the angle of the superior end and the straightness of the kidney whereas the third principal component is visually related to the kidneys’s dimensions in terms of the length and width. The fourth principal component appeared to be associated with kidney’s length and thickness. The remaining principal components are difficult to visually interpret.

### Survival analysis

We assessed whether the S2S distances had predictive value for the three disease outcomes diagnosed after the imaging visit. Here we identified 247 participants with CKD of which 59% were male, aged between 68.6 ± 6.5 years (mean ± SD) and a BMI between 16.4 and 48.9 kg/m^2^, 181 participants with T2D of which 61% were male, aged 65.4 ± 7.6 years with a BMI between 19.5 and 47.9 kg/m^2^ and finally, 733 participants with hypertension (56.2% male; aged 65.8 ± 7.1 years; BMI from 17.4 to 53 kg/m^2^). We created a model for each disease outcome adjusting for age, sex, ethnicity, body mass index, waist-to-hip ratio, alcohol intake frequency, smoking status, ibuprofen medication, sodium in urine, urea, kidney volume, and the first 4 PC scores derived from the PCA applied on the S2S distances (accounting for over 60% of the variation in S2S distances in all disease outcomes and for both kidneys), separately for each kidney (Fig. 4ii for the left kidney; Fig. 4iii for the right kidney).

We found that the third PC scores of the S2S distances were risk factors for CKD diagnosis in both the left kidney (0.74 [0.61–0.90]) and the right kidney (0.76 [0.63–0.92]). No PC scores of the S2S distances in both left and right kidneys were associated with T2D. This may be attributed to the relatively short follow-up period from the imaging visit (median 3.7 years, IQR 2.3 years) as well as the small number of incidences of T2D diagnosis (*N* = 181), resulting in reduced statistical power to detect significant associations.

We further found that increased right kidney volume was a risk factor for hypertension (1.60 [1.15–2.22]). The third PC score of the S2S distances for the left kidney was significantly positively associated with risk of hypertension (1.16 [1.04–1.29]) however, PC3 score of the S2S distances for the right kidney was significantly negatively associated with risk of hypertension (0.87 [0.79–0.96]).

## Discussion

In this study, we mapped local shape variations across the kidneys and determined how these variations were associated with anthropometric and disease traits. To achieve this we constructed surface meshes from kidney segmentations of 38,868 participants from the UK Biobank. Previous studies using similar SPM techniques have suggested that this is a useful technique in neuroimaging [14] and cardiac imaging [16], for mapping the associations between phenotypic and genetic variation in specific anatomical regions.

Measurements of the kidneys have been extensively explored using a variety of approaches from computed tomography angiography (CTA) [31], ultrasound [6], computed tomography (CT), and MRI [32], with assessments including measurements of the whole organ as well as the kidney sub-structures, and typically include measurement of size, length [33, 34], volume [3] and cortical thickness [35]. While these methods enhance our understanding of the kidneys in population-level studies, they do not capture specific morphological and regional variations that occur in the kidneys in response to specific conditions. The SPM method implemented in this study demonstrates significant regional variations that can be captured in each participant’s kidney shape.

Age was significantly associated with a decrease in S2S distances observed in the inferior and superior areas of both kidneys and was positively associated with S2S distances in the anterior and posterior areas of the kidneys. Previous studies investigating the kidney sub-structures of potential kidney donors found that cortical volume decreases with age and increases in medullary volume, with a slight difference in females [3]. Other studies investigating the parenchymal and total kidney volume of healthy individuals, found that kidney volume and length increased up to middle age followed by a continuous decrease in men and slowly decreased in women, suggesting some progressive loss of functional nephrons [36]. In our analysis, we found a similar decline in S2S distances with age for both sexes. However, future work will help to shed light on the shape variations from the kidney sub-structures such as the cortex and medulla.

We further found statistically significant positive associations between S2S distance and both BMI and WHR, confirming previous associations between BMI and kidney measurements [37]. Lifestyle factors, including alcohol consumption and smoking, also demonstrated positive associations with S2S distances, revealing notable shape variations. Increased kidney volume has been linked to smoking [3], and both smoking and heavy drinking have been associated with kidney dysfunction and various health risks [38, 39].

The presence of CKD was associated with reduced S2S in both kidneys. From previous sonographic measurements of the kidneys, it has been shown that a reduction in kidney volume can be used as an indicator of CKD [40] enabling the use of kidney size for the prediction of kidney function in healthy populations as well as patients with CKD [41, 42]. Participants with T2D and hypertension exhibited significant variations in S2S distance, consistent with reports that non-insulin dependent diabetics develop enlarged kidneys with hyperfiltration [43]. However, existing literature on the relationship between hyperfiltration and hypofiltration and kidney volume is unclear, showing both diabetes and hypertension being associated with hyperfiltration [44] while others reported no significant association between kidney volume and diastolic blood pressure [3]. Unlike conventional kidney measurements, which report a broader relationship with markers of CKD, diabetes and hypertension [45, 46], we have demonstrated that there is a strong and distinct regionality of the kidney shape in the face of these diseases, with simultaneous inward and outward adaptation. These findings emphasise the significance of utilising morphometric measurements to capture both global and local changes in the kidney morphology.

Interaction models within disease groups showed a stronger relationship between age and S2S distances in both kidneys with inward shape variation for all disease outcomes. More specifically our findings demonstrate that the added effect of age and CKD may further explain the variations in the kidneys’ S2S distances. This could be explained by the loss of nephrons, which lead to the progressive loss of kidney function as CKD progresses [47]. It is worth noting that due to the clinical heterogeneity of our current clinical cohort (CKD, T2D and hypertension), in terms of time of diagnosis and medication, as well as the possibility of collider bias or reverse confounding, it is not possible to identify causal mechanisms for the observed results. Despite this, our findings contribute to a better understanding of the complex relationship between kidney morphology and various factors.

We further explored variations in kidney morphometry by performing PCA on the full cohort. Our findings show that the kidney shape variations are heavily biased by organ size, angle, straightness, length-to-width ratio and length-to-thickness ratio, which are in line with previous findings [11]. Further work is needed to condense the entire coordinate matrix or deformation conventional matrix into most distinct modes to categorise population variations, which could be used in genetic association studies [48, 49].

We also investigated the risk of future disease outcomes adjusted for relevant anthropometric variables, kidney volumes as well as kidney S2S distances. Our findings revealed contrasting associations between the SSA-based S2S variations corresponding to the length and width of the kidneys with risk of hypertension. Specifically, we observed a significant positive association in the left kidney but a negative association in the right kidney. This difference may relate to the anatomical variations that predispose to atheroma in the renal artery which may then contribute to the associations with hypertension. This discrepancy may be also attributed to the significant anatomical differences in kidney length and width between the left and right sides. A previous study utilising sonographic measurements of renal size have also reported such disparities [50]. They also suggest that these differences may be attributed to the greater spatial capacity in the left kidney and a shorter length of the left renal artery compared to the right renal artery, resulting in increased blood flow and potentially an increase in kidney volume. We further demonstrated that variations corresponding to the length and width in the left and right kidney were associated with incidents of CKD. Previous studies measuring kidney size by ultrasonography, reported that kidney length and volume were correlated with eGFR levels in the elderly, however they report that kidney length had lower specificity in predicting kidney dysfunction [42]. Other studies investigating the accuracy of sonographic kidney measurements to detect kidney impairment and histological change, reported that kidney length to height ratio weighted for kidney echogenicity was able to detect kidney dysfunction [50]. Although these studies report the usefulness of the sonographic kidney measurements in detecting loss of kidney function, our study highlights the importance of the SSA-based shape features and the potential of integrating morphological features with clinical outcomes to enhance our understanding of the risk of future disease outcomes and prognosis.

Our study has limitations. The UK Biobank is a large cross-sectional study that is subject to selection bias with a “healthier” cohort than the wider UK population, who are predominantly of European ancestry, excludes younger participants and potentially more severe cases [51, 52]. However, it has been shown that risk factor associations are likely to be generalisable [53]. Furthermore, the current study is constrained by the limited resolution of the UK Biobank imaging acquisition which could lead to reduced sensitivity in detecting subtle morphological changes or structural variations. Despite this limitation, our analysis was still able to capture significant morphological variations in the kidneys in response to anthropometric traits and clinical conditions. Nevertheless, it is plausible that higher resolution imaging could enhance our analysis and show more significant associations. Moreover, it is essential to acknowledge that the true association between variables may not be linear in the model parameters. Nonlinear models might offer a better fit to the data in such cases. Nonetheless, the advantages supporting the use of a linear model encompass its simplicity, the ease with which it can be designed and adjusted for various factors, making it a commonly used tool in the field of biostatistics. Another potential limitation of this study is that to ensure sufficient numbers of participants in the CKD group, we included both participants based on CKD diagnosis codes reported at the imaging visit and participants based on eGFR levels taken at the initial assessment visit which precedes the imaging visit. Also, although albumin/creatinine ratio (ACR) is widely used to assess kidney function and diagnose diseases such as CKD, cardiovascular disease and diabetes [54, 55], this parameter was not used in this study as urine albumin was only available for 28% of the UK Biobank imaging cohort [56, 57]. Furthermore, this study has only a 3.7 years follow-up period since the imaging visit, which may limit the power of time-to-event studies. Additional longitudinal measurements will be required to assess age-related changes in disease cohorts.

## Conclusions

Our findings align with existing literature and can serve as a proof of principle, highlighting the potential scientific and clinical significance of shape analysis techniques. We found statistically significant associations between S2S distances and disease outcomes including CKD, T2D and hypertension. While the S2S distances provide important insights into kidney shape variations in relation to disease states, further research may be needed to fully establish their clinical implications and utility in a broader clinical population. We also identified significant associations between the SSA-based shape features corresponding to the size, length and width, and future events of disease outcomes. Therefore, the application of SPM and SSA-based shape analysis is feasible for improving our understanding of the variations in kidney shape associated with disease outcomes and their prediction. These techniques will benefit future research in population-based cohort studies, in identifying associations between physiological, genetic and environmental effects on structure and function of the kidneys as well as the kidney sub-structures such as cortex and medulla.

### Supplementary Information

**Additional file 1:** **Table S1. **Summary of the codes used to define disease. **Table S2. **Summary statistics (mean ± standard deviation, minimum and maximum values) for continuous variables and counts for discrete variables of the 200-participant gender-balanced cohort for the template construction. BMI: body mass index, WHR: waist-to-hip ratio, SBP: Systolic blood pressure, DBP: Diastolic blood pressure, eGFR: estimated glomerular filtration rate. **Table S3. **Summary statistics (mean ± standard deviation, minimum and maximum values) for continuous variables and counts for discrete variables in the full cohort (*N*=38,868). BMI: body mass index, WHR: waist-to-hip ratio, SBP: Systolic blood pressure, DBP: Diastolic blood pressure, eGFR: estimated glomerular filtration rate. **Table S4. **Standardised and unstandardised (raw) regression coefficients ($\widehat{\beta }$) for disease covariates in the MUR model for the left kidney and right kidney (*N*=38,868). The total area has been split into areas of positive and negative associations. The standardised regression coefficients are presented as median (interquartile range - IQR) across all vertices of the left and right kidney surfaces for the vertices with statistically significant associations. The unstandardised regression coefficients are based on the standardised coefficients and the median standard deviation of the S2S distances across all vertices (SD for the S2S distances is 3.02 mm for the left kidney and 2.95 mm for the right kidney). **Table S5. **Significance areas for covariates in the MUR model for the anthropometric variables of the model for the left kidney for each gender (18,855 males and 20,013 females). The total area has been split into areas of positive and negative associations. The standardised regression coefficients ($\widehat{\beta }$) are presented as median (interquartile range - IQR) across all vertices of the left kidney surface and the significance areas as a percentage (%) of the vertices with statistically significant associations. **Table S6. **Significance areas for covariates in the MUR model for the anthropometric variables (*N*=38,868) of the model for the right kidney for each gender (18,855 males and 20,013 females). The total area has been split into areas of positive and negative associations. The standardised regression coefficients ($\widehat{\beta }$) are presented as median (interquartile range - IQR) across all vertices of the left kidney surface and the significance areas as a percentage (%) of the vertices with statistically significant associations. **Figure S1. **Flow diagram of the study population used in this study (*N* = 38.868) of which 1,134 were diagnosed with CKD, 2,054 with T2D and 14,113 with hypertension. CKD: Chronic kidney disease; T2D: Type-2 diabetes. **Figure S2. **Average template mesh construction. Dixon MRI volumes from UK Biobank abdominal protocol (left) are used to produce subject-specific 3D kidney segmentations (middle), then images are registered to a common space and combined to produce average kidney template meshes. **Figure S3. **Flow diagram for the mass univariate regression (MUR) analysis of three-dimensional phenotypes. The phenotypes are used to construct the linear regression model. MUR analysis produces parameter estimates ($\widehat{\beta }$) and their null distribution via permutation. Threshold free cluster enhancement (TFCE) is applied to the $t$-statistics from the regression analysis to produce a significance threshold. The associated TFCE-derived $p$- values are corrected for multiple comparisons and mapped onto the kidney’s mesh for visualisation. This diagram was modified from [4]. **Figure S4. **Density plots showing the participants with CKD that are diagnosed by doctor, shown in red (*N* =793) and selected by eGFR below 60 ml/min/1.73 m², shown in blue (*N*=466), across eGFR levels. Means for each CKD are shown in dashed lines. **Figure S5. **Density plots showing the participants with hypertension (*N*=14,113) across blood pressure readings. The thresholds applied are for systolic blood pressure (SBP) ≥ 140 mmHg shown in blue solid line and diastolic blood pressure (DBP) ≥ 90 mmHg shown in red solid line. Means for each blood pressure reading are shown in dashed lines. **Figure S6. **Histograms showing the statistically significant regression coefficients across the vertices (~4,000) of the left kidney for each covariate in the model on the full cohort (*N* = 38,868) with positive associations in red and negative associations in blue. Beta coefficients are provided with units in standard deviations for each covariate. **Figure S7. **Histograms showing the statistically significant regression coefficients across the vertices (~4,000) of the right kidney for each covariate in the model on the full cohort (*N* = 38,868) with positive associations in red, negative associations in blue. Beta coefficients are provided with units in standard deviations for each covariate. **Figure S8. **Histograms showing the statistically significant regression coefficients across the vertices (~4,000) of the left kidney for each covariate in the model on the full cohort (*N* = 38,868) separated by gender with positive associations in red, negative associations in blue, female (*N* = 20,013) in light colour and male (*N* = 18,855) in darker colour. Beta coefficients are provided with units in standard deviations for each covariate. **Figure S9. **Histograms showing the statistically significant regression coefficients across the vertices (~4,000) of the right kidney for each covariate in the model on the full cohort (*N* = 38,868) separated by gender with positive associations in red, negative associations in blue, female (*N* = 20,013) in light colour and male (*N* = 18,855) in darker colour. Beta coefficients are provided with units in standard deviations for each covariate. **Figure S10. **Three-dimensional statistical parametric maps (SPMs) of kidney morphology, projections are anterior (left plots) and posterior (right plots) views for both left (L) and right (R) kidneys in both anterior (left) and posterior (right) views. The SPMs show the local strength of association for each covariate in the model with S2S distances on the male cohort (*N* = 18,855). Yellow contour lines indicate the boundary between statistically significant regions (*p* < 0.05) after correction for multiple testing, with positive associations in bright red and negative associations in bright blue. Regression coefficients are shown with units in standard deviations for each covariate. **Figure S11. **Three-dimensional statistical parametric maps (SPMs) of kidney morphology, projections are anterior (left plots) and posterior (right plots) views for both left (L) and right (R) kidneys in both anterior (left) and posterior (right) views. The SPMs show the local strength of association for each covariate in the model with S2S distances on the female cohort (*N* = 20,013). Yellow contour lines indicate the boundary between statistically significant regions (*p* < 0.05) after correction for multiple testing, with positive associations in bright red and negative associations in bright blue. Regression coefficients are shown with units in standard deviations for each covariate. **Figure S12. **The percentage of shape variation explained by the first ten modes of PCA for the kidneys of the full cohort (*N* = 38,868). **Video S1. **The first 4 modes of shape variation for the kidneys of the full cohort (*N* = 38,868). The mean shape and the shape at the +/- 3 standard deviations are displayed for each mode showing the S2S distance change in mm. The right kidney is shown on the left side and the left kidney on the right side of the video.

## Data Availability

The UK Biobank resource is available to bona fide researchers for health-related research in the public interest. All researchers who wish to access the research resource must register with UK Biobank by completing the registration form in the Access Management System (AMS—https://bbams.ndph.ox.ac.uk/ams/).

## Abbreviations

CKD: Chronic kidney disease
T2D: Type 2 diabetes
BMI: Body mass index
WHR: Waist-to-hip ratio
eGFR: Estimated glomerular filtration rate
MUR: Mass univariate regression
TFCE: Threshold-free cluster enhancement
SPMs: Statistical parametric maps
SSA: Statistical Shape Analysis
PCA: Principal component analysis
S2S: Surface-to-surface
